# Targeting SIRT2 in Aging-Associated Fibrosis Pathophysiology

**DOI:** 10.14336/AD.202.0513

**Published:** 2025-07-05

**Authors:** Yongjiao Huang, Wei He, Yingting Zhang, Zhihui Zou, Longchuan Han, Jing Luo, Yunqiu Wang, Xinxin Tang, Yue Li, Yuhan Bao, Ying Huang, Xi-Dai Long, Yinkun Fu, Ming He

**Affiliations:** ^1^Department of Pathophysiology, Key Laboratory of Cell Differentiation and Apoptosis of Ministry of Education, Shanghai Frontiers Science Center of Cellular Homeostasis and Human Diseases, Shanghai Jiao Tong University School of Medicine, Shanghai, China.; ^2^Department of Pathology, The Affiliated Hospital of Youjiang Medical University for Nationalities, Baise, China.; ^3^School of Basic Medicine, DeHong Vocational College, Dehong, Yunnan, China.; ^4^School of Basic Medicine, Kunming Medical University, Kunming, China.; ^5^Toxicology Department,Sichuan Center For Disease Control and Prevention, Chengdu, Sichuan, China.; ^6^Department of Biomedical Sciences and Synthetic Organic Chemistry, University College London, United Kingdom.; ^7^Department of Biological Sciences, University of Auckland, Auckland, New Zealand.; ^8^Clinicopathological Diagnosis & Research Center, the Affiliated Hospital of Youjiang Medical University for Nationalities, Guangxi, China.

**Keywords:** Aging, SIRT2, Liver fibrosis, Renal fibrosis, Cardiac fibrosis

## Abstract

Aging is a complex biological process that involves multi-level structural and physiological changes. Aging is a major risk factor for many chronic diseases. The accumulation of senescent cells changes the tissue microenvironment and is closely associated with the occurrence and development of tissue and organ fibrosis. Fibrosis is the result of dysregulated tissue repair response in the development of chronic inflammatory diseases. Recent studies have clearly indicated that SIRT2 is involved in regulating the progression of fibrosis, making it a potential target for anti-fibrotic drugs. SIRT2 is a NAD^+^ dependent histone deacetylase, shuttling between nucleus and cytoplasm, and is highly expressed in liver, kidney and heart, playing an important role in the occurrence and development of aging and fibrosis. Therefore, we summarized the role of SIRT2 in liver, kidney and cardiac fibrosis during aging.

## 1. Introduction

Fibrosis is a pathological process characterized by excessive accumulation of extracellular matrix (ECM) components. This accumulation results in progressive structural remodeling of almost all tissues and organs, which may ultimately lead to death [1].The occurrence and progression of fibrosis is closely related to aging, and its morbidity and mortality gradually increased when aging [2].However, fibrosis does not always herald a negative outcome; on the contrary, it plays a protective role, acting as a crucial survival mechanism in the body. For instance, acute injuries can trigger fibrosis to form a stable scar structure, maintaining the organ’s regular function [3]. When harmful stimuli become chronic or when anti-fibrotic signals are suppressed, cellular fibrotic effectors become overstimulated. This causes a shift in equilibrium between ECM protein degradation and synthesis towards the latter, which expands fibrosis and possibly leads to organ failure [4].A significant increase in the number of myofibroblasts during fibrosis will lead to structural abnormalities and organ dysfunction, inevitably exacerbating the disease progression. At present, artificial or organ replacement therapy is the only method [5].Therefore, it is urgent to study the pathogenesis of age-related diseases and fibrosis.

SIRT2 is a NAD^+^ dependent deacetylase that can control multiple metabolic pathways and aging, with highly conserved NAD^+^ binding and catalytic domains [6] SIRT2 is highly expressed in the central nervous system, adipose tissue, liver, and heart, with subcellular localization in the cytoplasm, nucleus, and mitochondria [7]. As the earliest discovered member of the Sirtuin family, SIRT2 plays an important role in maintaining metabolic homeostasis in various physiological processes, including inflammation, oxidative stress, mitochondrial function, as well as lipid and alcohol metabolism [8]. The involvement of SIRT2 regulation in inflammation has been extensively reported: SIRT2 deacetylates p65 to regulate the expression of specific NF-κB dependent genes, SIRT2 deacetylates NLRP3 inflammasomes to reverse age-related inflammation and in vitro insulin resistance [9]. Meanwhile, SIRT2 inhibitors are also used for disease treatment. Research has found that the SIRT2/ERK/c-MYC axis plays a crucial role in promoting liver fibrosis, and SIRT2 inhibitors are a potential new strategy for treating liver fibrosis and cirrhosis. In addition, SIRT2 inhibition alleviates fibroblast activation and renal tubulointerstitial fibrosis [10], while SIRT2 inhibitors can alleviate inflammation and suppress idiopathic pulmonary fibrosis [11]. Therefore, SIRT2 is expected to become a new target for preventing and treating fibrosis. This article will summarize the functions of SIRT2, with a focus on exploring its molecular mechanism in aging related organ fibrosis.

## 2. Aging and fibrosis

### 2.1 Aging

**Figure 1.**
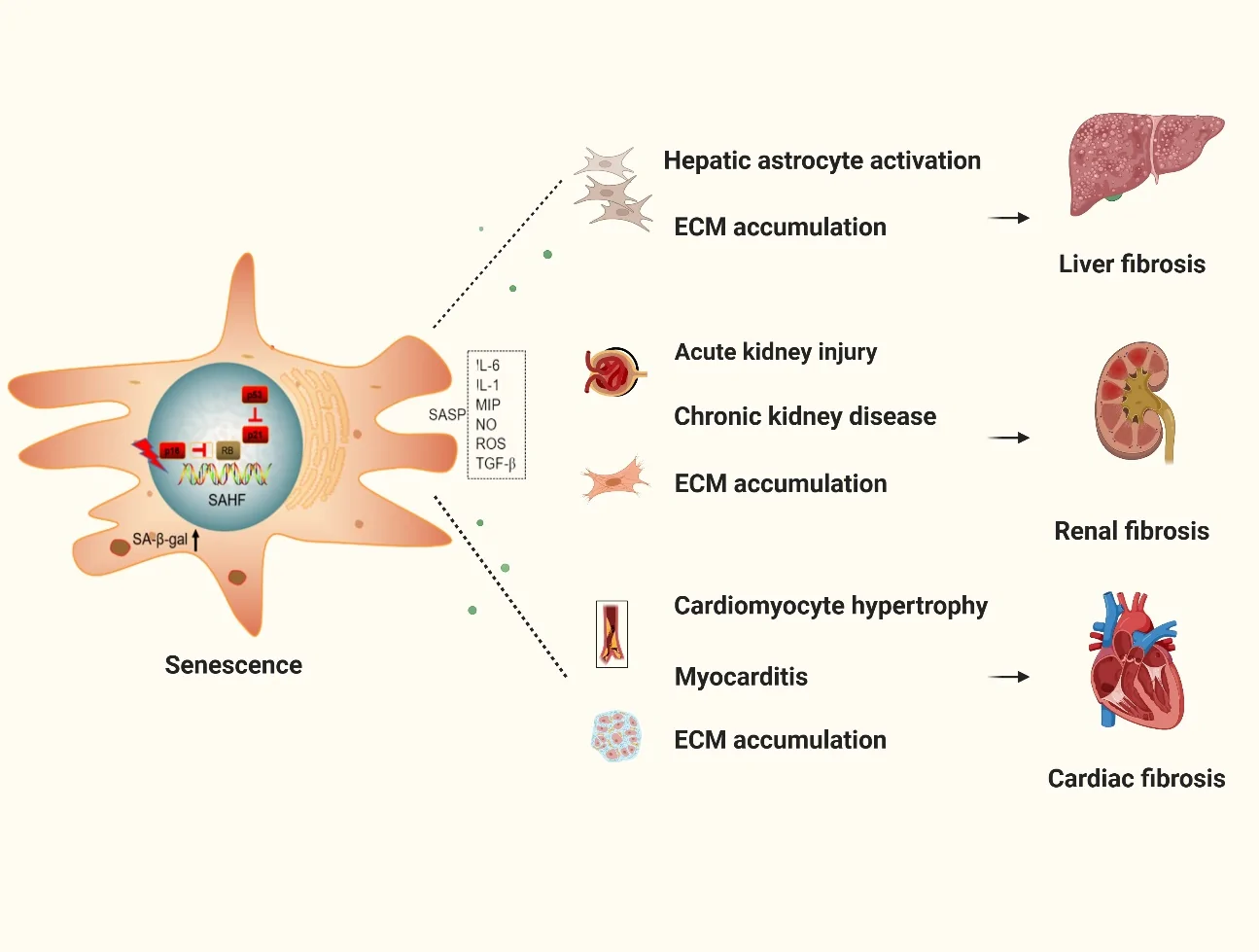
**Aging and fibrosis**. Aging is a complex biological process, and the expression of SA-β-gal and the increase of cyclin-dependent kinase inhibition molecules p53, p21 and p16 are key markers for evaluating whether cells are aging, and age-associated heterochromatin lesions and SASPs are also important molecular characteristics of aging. The accumulation of senescent cells changes the microenvironment, causing fibrosis in the liver, kidney, and heart.

Aging is a complex biological process, including structural and physiological changes at multiple levels. Key aging indicators include temporal accumulation of genomic instability, telomere attrition, epigenetic alternations, loss of proteostasis, and macroautophagy inactivation. At different degrees, the opposite antagonistic markers of aging are nutrient dysregulation, mitochondrial dysfunction, and cellular senescence. Combined markers occur when damages from fundamental and antagonistic hallmarks remain irreparable: stem cell depletion, altered intercellular communication, chronic inflammation, and dysregulation of gut microbes [12].Cellular senescence is a state of cell cycle arrest in response to the stimulation of DNA damage, telomere dysfunction, oncogene activation, and other conditions, resulting in an irreversible growth halt [13]. Significantly increased expression of senescence-associated β-galactosidase (SA-β-Gal) and cyclin-dependent kinase (CDK) inhibitory molecules p53, p21, and p16 are key markers for cell senescence evaluation. In aging cells, the number and size of lysosomes will increase. For example, lipofuscin, which is an aggregate of oxidized proteins, lipids, and metals, is associated with increased lysosomal mass and aging related SA-β-Gal activity. Among them, p53/p21 and p16/ retinoblastoma (RB) protein signaling pathways play an essential role in cell cycle arrest. The accumulation of aging cells leads to sustained hypophosphorylation of RB family proteins, inhibition of E2F transcription factors, downregulation of proliferation marker MKI67, and subsequent cell cycle arrest. This blockage is caused by various factors, including chromatin changes in E2F target genes and defects in ribosomal biology [14]. Furthermore, senescence-associated heterochromatin foci (SAHF) and senescence-associated secretory phenotype (SASP) are important molecular features of aging. SASP includes pro-inflammatory cytokines such as interleukin-6 (IL-6), Interleukin-1 (IL-1), interleukin-8 (IL-8), MCP-1, macrophage inflammatory proteins (MIP) fibronectin, ROS and nitric oxide. These factors alter the tissue microenvironment when inducing inflammation and adjacent cellular senescence in a paracrine manner [15]. Senescence is a major risk factor for numerous chronic diseases. Aging related diseases are often accompanied by fibrosis, and there is a complex connection between cellular aging and the occurrence of tissue and organ fibrosis [16].Aging involves the gradual loss of tissue function over time, and a major characteristic of aging is the accumulation of aging cells. Aging cells accumulate abnormally and continuously in the later stages of life, leading to age-related fibrosis. In addition, aging can lead to chronic inflammation, which is one of the most common age-related phenotypes. The ROS produced by dysfunctional mitochondria can also trigger an inflammatory response by activating nuclear factor kappa B [17] This leads to fibrosis and organ dysfunction, indicating a close correlation between fibrosis and aging. Aging related fibrosis occurs in various diseases, such as vascular disease, idiopathic pulmonary fibrosis, cardiac fibrosis, liver fibrosis, and chronic kidney disease. Long term abnormal accumulation of aging cells can produce SASP, in which TGF-β, IL-1β, and IL-6 enhance fibrosis and inflammatory response (Fig. 1).

### 2.2 Aging and liver fibrosis

Liver fibrosis is typically triggered by damage to liver cells or biliary tract cells, followed by an inflammatory response. This response activates hepatic stellate cells (HSC) and promotes ECM accumulation. HSC is a major regulator of fibrosis pathogenesis and can be activated via various cell signaling pathways and cytokines. The accumulation of senescent cells creates an inflammatory environment, culminating in liver fibrosis. The elimination of senescent cells can reduce liver fibrosis [18].Cell senescence and HSC proliferation occur during liver fibrosis [19], while accumulation of senescent bile duct cells has harmful effects on the bile duct through SASP, whereas HSC senescence acts as a countermeasure, limiting liver fibrosis progression [20]. SASP can induce cell aging and regulate fibrosis through the mechanism of TGF-β producing reactive oxygen species and DDR. TGF-β1 can promote the occurrence of liver fibrosis, whereas AMPK acts as a key molecule to prevent fibrosis. The activation of the AMPK/TGF-β1 pathway can inhibit liver fibrosis [21]. IL-22 offers protection against liver fibrosis by mediating HSC senescence via p53 pathway [22].

### 2.3 Aging and renal fibrosis

Renal fibrosis, characterized by abnormal accumulation of ECM in the renal interstitium, is a worsening process of various end-stage renal diseases. As age increases, the incidence of renal fibrosis dramatically rises, coinciding with the senescence of renal tubule cells in the process of renal fibrosis [23].Acute kidney injury causes the renal cell cycle to stagger in the G2/M phase, subsequently releasing pro-fibrotic cytokines, which promote the development of renal fibrosis [24]. Recurrent episodes of acute kidney injury can lead to chronic kidney disease (CKD), marked by reduced regenerative capacity, microvascular damage, metabolic changes, oxidative stress, and inflammation, eventually leading to fibrosis. TGF-β1 is a major regulator of myofibroblast differentiation in fibrosis [25]. Senescent renal epithelial cells produce multiple components of the SASP. TGF-β1 induces fibrosis while inhibiting the regeneration of injured renal tubules [26]. Klotho-derived peptide 1 (KP1) prevents renal fibrosis by targeting TGF-β signaling pathway [27]

### 2.4 Aging and cardiac fibrosis

Cardiac fibrosis emerges as a sustained reparative response to both inflammation and aging, denoted by the excessive production and deposition of scar tissue. Cardiac fibrosis is primarily activated by the conversion of ECM produced by fibroblasts into myofibroblasts by TGF-β. Concurrently, the aging heart exhibits distinct histological and biochemical traits, including proliferation of muscle nuclei, increased muscle cell volume, connective tissue accumulation, and increased lipofuscin levels [28]. Aging-stimulated fibroblasts exhibit increased collagen deposition and endovascular matrix thickening. On the other hand, the local angiotensin system plays an important role in age-associated vascular remodeling, with genes associated to arterial stiffness and pulse pressure predominantly regulated by the renin-angiotensin system [29]. Excessive deposition of ECM, under the regulation of resident fibroblasts, modulates the balance between collagen synthesis and degradation and provides structural support and tissue repair. Aging-associated cardiac fibrosis is characterized by the progressive loss of muscle cells through necrosis and apoptosis, accompanied by increased myocardial collagen content, resulting in cardiomyocyte hypertrophy and myocardial fibrosis [30].The progressive cardiomyocyte hypertrophy, inflammation, and the progressive development of cardiac fibrosis are the hallmarks of cardiac aging [31].

## 3. SIRT2 and Sirtuin protein family

The Sirtuin protein family comprises a class of NAD-dependent deacetylases with highly conserved NAD-binding and catalytic domains, ubiquitous in prokaryotes and all eukaryotes [32].Sirtuins are associated with metabolism, aging, and age-associated diseases. The human Sirtuin gene family consists of seven members, each of which contains a conserved core domain, with some variants also presenting additional N-terminal or C-terminal. Functionally, Sirtuins are categorized into four classes: Class I includes SIRT1, SIRT2, and SIRT3; SIRT4 represents Class II; SIRT5 is Class III; and SIRT6 and SIRT7 are class IV [33]. Despite their diversity in tissue specificity, sub-cellular localization, enzyme activity, and targets [34], Sirtuins uniformly exert critical function across cells, tissues, organs, and systemic levels.

**Table 1:** Positioning and substrate of the Sirtuin family.

Distribution and Targets of Sirtuins			
**Subtype**	Subcellular localization	Substrate	Main distribution
**SIRT1**	Nucleus [35], cytoplasm [36]	TP53 [37], FoxO [38], NF-κB [39], p53 [40]	Kidney [41], skeletal muscle, heart [42]
**SIRT2**	Nucleus, cytoplasm [43]	P53 [44], P73 [45], HNF4 [46], STAT3 [47], MARCKS [48], PRDX-1 [49], αTubulin [50], SMC1A [51], HIDTONEH3, P300 [52], C/EBPβ [53]	Central system [54], adipose tissue [55], liver, heart, kidney [56]
**SIRT3**	Mitochondrial matrix [57], cytoplasm [58]	Foxo3a [59], PPID [60], ACADL [61],HMGCS2 [62] NDUFA9 [63],ATP5a [64],LATS1 [65]	Liver, brown fat [66], heart, muscle [67]
**SIRT4**	Mitochondria [68], cytoplasm, nucleus [69]	LATS1 [65],SCFD1 [70],OPA1 [71]	Vascular smooth muscle cells and skeletal muscle cells [72]
**SIRT5**	Mitochondria, cytoplasm [73]	PKM2 [74]	Brain, heart, liver, kidneys, muscles, testicles [75]
**SIRT6**	Nucleus [76]	CtIP [77],PARP1 [78],KAP1 [79],GCN5 [80],FoxO3a [81],NPM1 [82]	Heart, kidney [83]
**SIRT7**	Nucleus [84]	GABPβ1 [85],mTOR [86],DDB1 [87]	Brain [88], Kidney [89], testicles, heart [90]

Now we are focusing on SIRT2, the first discovered member of the Sirtuin family. SIRT2 has been implicated in various cellular processes, including myelination, cell cycle, genomic stability, longevity, autophagy, immune response, neurodegeneration, and fibrosis [91]. As emerging research hotspots, 12 SIRT2 inhibitors have entered preclinical studies [92] (Table 2). SIRT2 is highly enriched in metabolically active tissues, notably the liver and kidney [93]. Serving as a major metabolic regulator, SIRT2 can deacetylate a range of proteins. Beyond its recognized role in aging regulation and neurodegenerative diseases [94], SIRT2 has also shown an unusual function in the development of fibrosis in the liver, kidney, and heart. Our recent studies showed SIRT2 plays pivotal roles in aging-associated biological process, including chronic inflammation and bone-liver crosstalk. Meanwhile, accumulated studies showed that the role of SIRT2 in cardiovascular diseases is gradually being unveiled, especially in aging-induced vascular remodeling and age-associated cardiac dysfunction. These studies coincidentally demonstrate that SIRT2 may be a novel target for prevention and treatment of ageing associated diseases. This article mainly focusses on the roles of SIRT2 in aging-associated fibrosis of different organs.

**Table 2:** SIRT2 inhibitors and therapeutic effects.

SIRT2 inhibitors and therapeutic effects	
**Drug name**	Therapeutic field
**Ro-318220**	Immunosuppressant
**Gambino**	Tumorolytic drugs
**AGK2**	Anti-parkinson disease
**Methaflutein**	Antitumor antibiotic
**Tenovan-6**	Tumorolytic drugs
**Shalmite**	Tumorolytic drugs
**AK7**	Huntington's disease, Parkinson's disease
**Tenovan-1**	Tumorolytic drugs
**PEP-1-SIRT2**	Edema and inflammation
**886549**	Tumorolytic drugs
**781090**	Neurodegenerative diseases; antidepressant
**BPDZ-711**	Oncolytic drugs, antihypertensive drugs, diabetes drugs, insulin, ischemia

## 4. SIRT2-mediated liver fibrosis during aging

**Figure 2.**
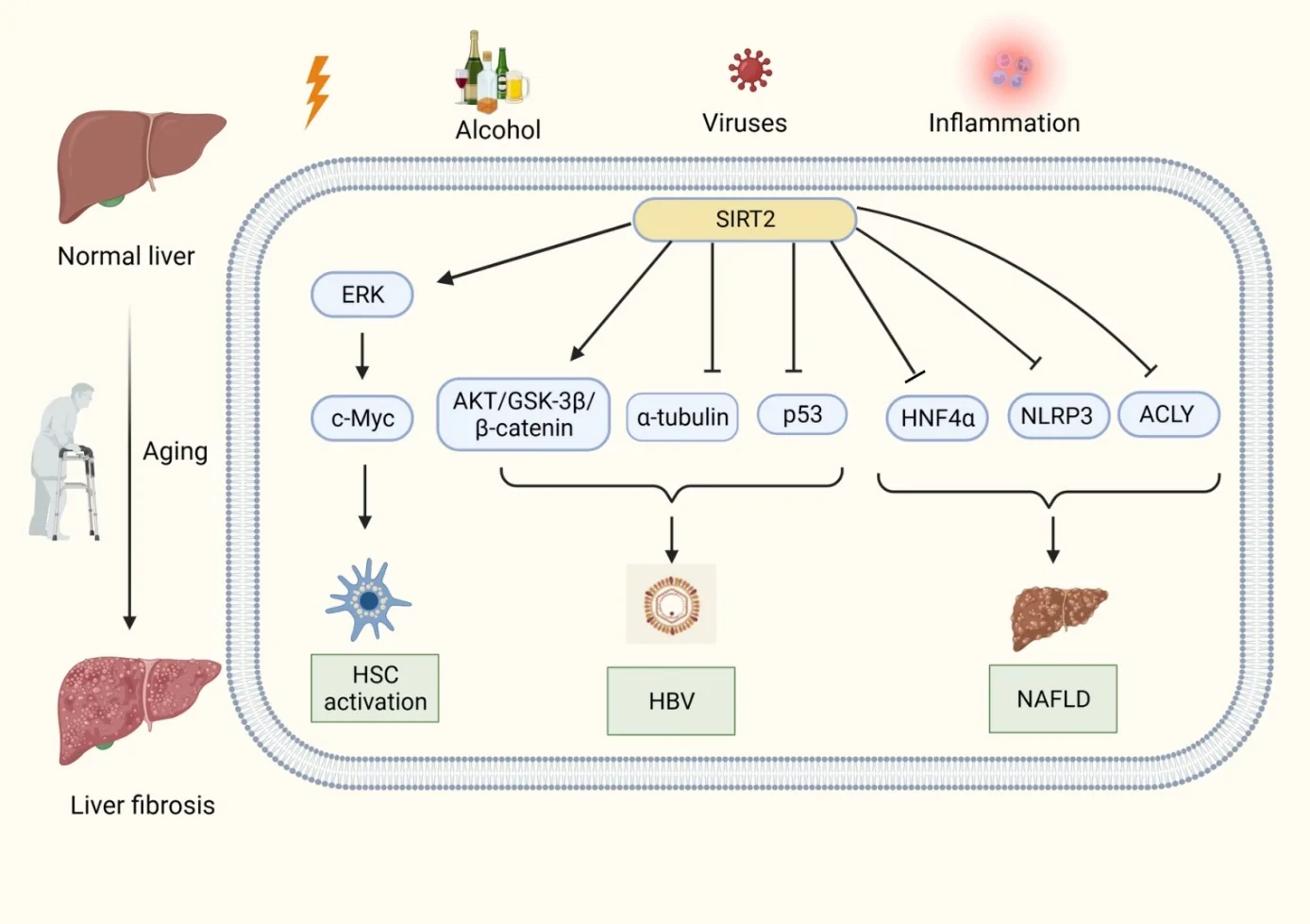
**SIRT2-mediated liver fibrosis**. Aging increases the risk of chronic liver disease. Alcohol, viruses, and inflammation are major contributors to liver injury that promotes the progression of chronic liver disease to severe fibrosis. SIRT2 can affect fibrosis by acting on different signaling pathways. On the one hand, deacetylation of SIRT2 can activate the AKT / GSK-3β / β-catenin signaling pathway and thus affect the phosphorylation of FOXO to promote HBV replication, and at the same time, HBx can increase the expression of SIRT2 through the targeting of promoters to promote HBV transcription and replication; on the other hand, SIRT2 can promote the replication of HBV through the inhibition of α-tubulin and P53. On the other hand, SIRT2 inhibits HBV replication by suppressing α-tubulin and P53.Meanwhile, SIRT2 regulates the development of NAFLD through deacetylating HNF4α, ACLY, and NLRP3. Moreover, SIRT2 promotes HSCs activation and liver fibrosis by promoting ERK signaling pathways. NAFLD: non-alcoholic steatohepatitis; HSC: hepatic stellate cells.

The liver, an important metabolic organ, plays an integral role in deoxidation, glucose storage, and bile acid secretion. So, an abnormal loss of liver will affect normal physiological responses. Liver fibrosis arises as a consequence of prolonged liver damage caused by various factors, such as alcohol consumption, non-alcoholic steatohepatitis (NASH), viral hepatitis (hepatitis B (HBV) and C), autoimmune hepatitis, non-alcoholic steatohepatitis (NAFLD) and cholestatic liver disease [95]. The Sirtuin family is considered to be closely associated with the aging process, and in most cases, its activation will prevent the aging process [96]. SIRT2 is a silencing regulatory protein that is highly expressed in the liver. Studies have shown that SIRT2 is expressed in human fibrotic tissue and over-expressed SIRT2 promotes liver fibrosis [97]. Contrarily, SIRT2 up-regulation may contribute to the maintenance of aging, and SIRT2 may act as a novel age-associated marker, including age-associated gene expression through deacetylation of histone H4 / NF-κB [98]. SIRT2 deficiency leads to fibrosis, signaling an adverse outcome in liver disease progression which can be considered as a precursor pathology of cirrhosis and hepatocellular carcinoma. Aging-associated SIRT2 alternations correlate with HSC activation in viral hepatitis, non-alcoholic fatty liver disease, and fibrosis onset. Importantly, SIRT2 modulates fibrosis via different signaling pathways (Fig. 2). Aging itself is the main risk factor promoting the development of different stages of chronic liver disease (inflammation, non-alcoholic liver disease, and fibrosis). The incidence rate of chronic liver disease increases sharply with age and has a worse prognosis. The aging of different cells plays different roles in the progression of liver fibrosis. Although the aging of liver cells and bile duct cells is related to the development of fibrosis, the aging of hepatic stellate cells leads to the regression of fibrosis. Maintaining the balance between aging cells is crucial. Intermittent anti-aging therapy should be used in combination with anti-aging drugs to better treat liver fibrosis.

### 4.1. The role of SIRT2 on the activation of hepatic stellate cell during aging

Hepatic stellate cells (HSCs) are the main source of ECM in liver fibrosis. This is a group of mesenchymal cells located in the hepatic sinus space (Dise space), surrounded by liver cells and sinusoidal endothelial cells. The main function is to secrete laminin, proteoglycans, and type IV collagen to form a basement membrane like structure. Static HSCs begin to proliferate and undergo transdifferentiation into contractile myofibroblasts, responding to paracrine stimuli from neighboring cell types including Kupffer cells, liver cells, platelets, white blood cells, and sinusoidal endothelial cells. Kupffer cells can stimulate the activation and proliferation of HSCs through the action of cytokines, and static HSCs store retinoids in lipid droplets in the cytoplasm. Retinol like derivatives play an important role in tissue homeostasis and are involved in proliferation, differentiation, and immune signaling. After activation, HSCs lose their ability to store retinoids and begin to proliferate, contract, and upregulate the synthesis of ECM components and ECM modifying enzymes. Activated HSCs also produce fibroblasts, growth factors, and morphogenetic proteins, which in turn regulate and affect tissue structure. The expression of α-smooth muscle actin (α-SMA) is the most prominent marker for identifying activated HSCs. It changes the cytoskeleton to drive cell movement and contraction and is believed to regulate signal transduction during wound healing. The fiber components produced by activated HSCs are mainly composed of type I and III collagen, as well as ECM proteins [99].

On one hand, SIRT2 enhances the activation of hepatic stellate cells. Inhibiting SIRT2 by using drug inhibitors or shRNAs significantly suppresses the production of type I collagen (COL1A1) and α-SMA in primary HSCs and HSC cell lines, both of which are markers of the stellate cells’ activation. C-MYC is crucial in regulating various biological processes, including cell division, apoptosis, cell growth, and angiogenesis [100], [101]. C-MYC also regulates the activation of HSCs [102]. In HSCs, SIRT2 binds to ERK and deacetylates to stabilize C-MYC. SIRT2 deficiency mice are less susceptible to the development of carbon tetrachloride (CCl4)/TAA-induced liver fibrosis [103]. On the other hand, cells that lack SIRT2 detected SASP, revealing an elevated expression of cell cycle retardation factor p21 and p53. The enhanced senescence of hepatocytes suggests that SIRT2 deficiency would lead to senescence of hepatocytes and stimulate ECM production by HSCs, culminating in fibrosis [104].

### 4.2. Effects of SIRT2 on HBV replication and transcription during aging

Epidemiological statistical analysis indicates that approximately 50-80% of HCC cases are caused by HBV infection globally [105]. HBV infection can promote hepatocyte carcinogenesis through direct or indirect mechanism [106]. Chronic HBV infection is a major contributor to cirrhosis, with SIRT2 regulating fibrosis by affecting HBV replication and transcription.

#### 4.2.1. SIRT2 promotes HBV replication by activating AKT

AKT, a downstream target of phosphatidylinositol 3-kinase (PI3K), is a major component in signal transduction pathways that regulate many cellular processes, including proliferation, differentiation, and survival [107]. HBV activates the PI3K-AKT pathway to inhibit apoptosis, promote the survival of infected cells, and facilitate viral replication [108].

SIRT2 physically interacts with AKT, which is essential for AKT activation: Inactivation of SIRT2 reduces insulin-mediated activation of AKT, while SIRT2 over-expression increases AKT activation along with its downstream targets [109]. SIRT2 deacetylation is also required for AKT activation [110]. In summary, SIRT2 promotes HBV replication through the AKT/GSK-3β / β-catenin signaling pathway. Moreover, PI3K /AKT pathway phosphorylates FOXO. FOXO1/3 is a significant regulator of aging and longevity, known to activate and up-regulate cell cycle inhibitors in HSC, leading to cell cycle arrest and inhibiting HSC proliferation, thus affecting fibrosis [111].

#### 4.2.2. SIRT2 deacetylation of α-tubulin increases HBV transcriptional activity

Microtubules (MTs) are ubiquitous cytoskeletal components that play a key role in various cellular processes, encompassing cell morphology, division, mobility, and intracellular transport [112]. The early post-fusion stages of viral entry into cells and infection require intact and dynamic microtubules, with the recombination of microtubule cytoskeleton that occurs during cellular senescence [113].

SIRT2 is a deacetylase of cytoplasmic α-tubulin. In HCC cells containing replicating HBV, the acetylation level of α-tubulin decreased, with HBV replication prompting an up-regulation of endogenous SIRT2. Consequently, α-tubulin deacetylation increases, with the over-expression of SIRT2 accentuating the transcriptional activity of HBV.

#### 4.2.3. SIRT2 collaborates with HBx to promote HBV replication and transcription

The HBV gene mainly encodes HBsAg, HBcAg, HBeAg, and HBxAg antigens. HBx is essential for HBV replication in vivo and significantly contributes to HCC development [114]. HBx can increase SIRT2 expression by targeting its promoter, thereby promoting HBV transcription and replication; HBx also enhances HCC cell migration and invasion in a SIRT2-dependent manner [115].

#### 4.2.4. SIRT2 enhances HBV replication and transcription by downregulating the expression of p53

P53 is a transcription factor that plays a central role in the aging process. P53 induces cell senescence, regulates cell cycle and mediates apoptosis [116]. SIRT2 enhances the activity of HBV EnI/Xp and EnII/Cp through the downregulating of p53 expression. This action amplifies the transcriptional activity of HBV cccDNA, thereby promoting HBV transcription and replication [117].

### 4.3. SIRT2 and the occurrence and development of NAFLD in aging

NAFLD is the most common chronic liver disease, with a global prevalence rate of 25.24% [118]. NAFLD not only makes patients susceptible to a range of liver diseases including liver bone disease, steatohepatitis, cirrhosis, ultimately leading to HCC, but also increases the risk of cardiovascular metabolic diseases [119]. Hepatic steatosis emerges as a result of excessive accumulation of lipids in the liver, indicating an early indicator of NAFLD [120]. Senescence is associated with cellular lipid metabolism. Lipid accumulation in hepatocytes promotes the further development of NAFLD. Animal experiments have shown that hepatocytes become aging via p16 and p21 pathways, correlating with heightened TAG [121]. Because non-alcoholic fatty liver disease is an important cause of liver cirrhosis and SIRT2 can be regulated through different signaling pathways of lipid metabolism, SIRT2 is also important for liver fibrosis.

#### 4.3.1. SIRT2 stabilize HNF4α expression

The liver-specific hepatic nuclear factor 4α (HNF4α) is a member of the nuclear receptor superfamily [122]. As the main regulator of liver-specific gene expression, HNF4α regulates lipid, amino acid and glucose metabolism [123]. Hepatic HNF4α is essential for maintaining lipid homeostasis and has a protective effect on NAFLD [124]. The accumulation of senescent cells generates reactive oxygen species (ROS) that stimulate HNF-4α expression through the ASK1-CREB pathway, thereby promoting ChREBP expression and adipogenesis in hepatocytes [125]. Animal experiments show that HNF4α promotes glucose synthesis in senescent hepatocytes [126]. HNF4α is a downstream target of SIRT2. SIRT2 can bind and deacetylate HNF4α to elevate the HNF4α protein level. Importantly, deacetylation of HNF4α by SIRT2 on Lys458 residues is necessary for its protein stability [127]. Therefore, targeting the SIRT2-HNF4α pathway may be a promising strategy for NAFLD treatment.

#### 4.3.2. SIRT2 damages ACLY stability

ATP-citrate lyase (ACLY) is a critical lipid-producing enzyme that regulates the initial steps of lipid biosynthesis. ACLY catalyzes the ATP-consuming reaction to generate acetyl-CoA from citrate, which is a key component in lipogenesis [128]. Db/Db mice with morbid obesity, fatty liver disease and type 2 diabetes [129] have up-regulated ACLY expression levels in the livers. ACLY regulates cellular senescence via AMPK and p53-dependent pathways [130]. The accumulation of senescent cells promotes hepatic fat deposit and steatosis, which promotes the development of NAFLD [131]. Thus, the high expression of ACLY might play an important role in NAFLD progression.

Over-expression of SIRT2 impairs ACLY acetylation at its 3K site (Lys-540, Lys-546, and Lys-554), depleting ACLY protein stability and weakening lipid accumulation in AML12 cells. SIRT2 alleviated liver steatosis in mice fed a high fat/high sucrose (HF/HS) diet [132]. The SIRT2/ ACLY axis involved in NAFLD progression could be a potential therapeutic target.

#### 4.3.3. SIRT2 deacetylates NLRP3 and inactivates the NLRP3 inflammasome

Aging leads to gradual impairment of homeostasis across genomic, cellular, tissue, and organism levels, amplifying chronic liver disease susceptibility, cellular aging and inflammation. Inflammation, known as a prime contributor to liver damage, can escalate liver steatosis to severe fibrosis [133]. During aging, activation of NLRP3 inflammasome mediates caspase-1 activity and induces maturation, secreting pro-inflammatory cytokines such as IL-1β and IL-18. Oxidative stress and lipid accumulation activate hepatic stellate cells and exacerbate fibrosis progression [134]. NLRP3 was found to be targeted by SIRT2 deacetylation to reverse aging-associated inflammation in the aging mouse model.

In addition, SIRT2-mediated C/EBP-β deacetylation in alcoholic liver disease presents a critical protective effect against ethanol-induced liver injury. C/EBP-β is associated with aging and the progression of NAFLD [135]. A number of key enzymes are regulated by the cdk4-C/ EBP-α-P300 pathway, which is critical for the development of age-dependent hepatic steatosis [136]. In conclusion, SIRT2 can participate in the pathogenesis of liver fibrosis during aging by activating HSC, promoting HBV replication and regulating lipid metabolism. SIRT2-specific inhibitors have also been shown to reverse liver fibrosis, which indicates targeting SIRT2 may become a potential strategy.

## 5. SIRT2 affects renal fibrosis during aging

**Figure 3.**
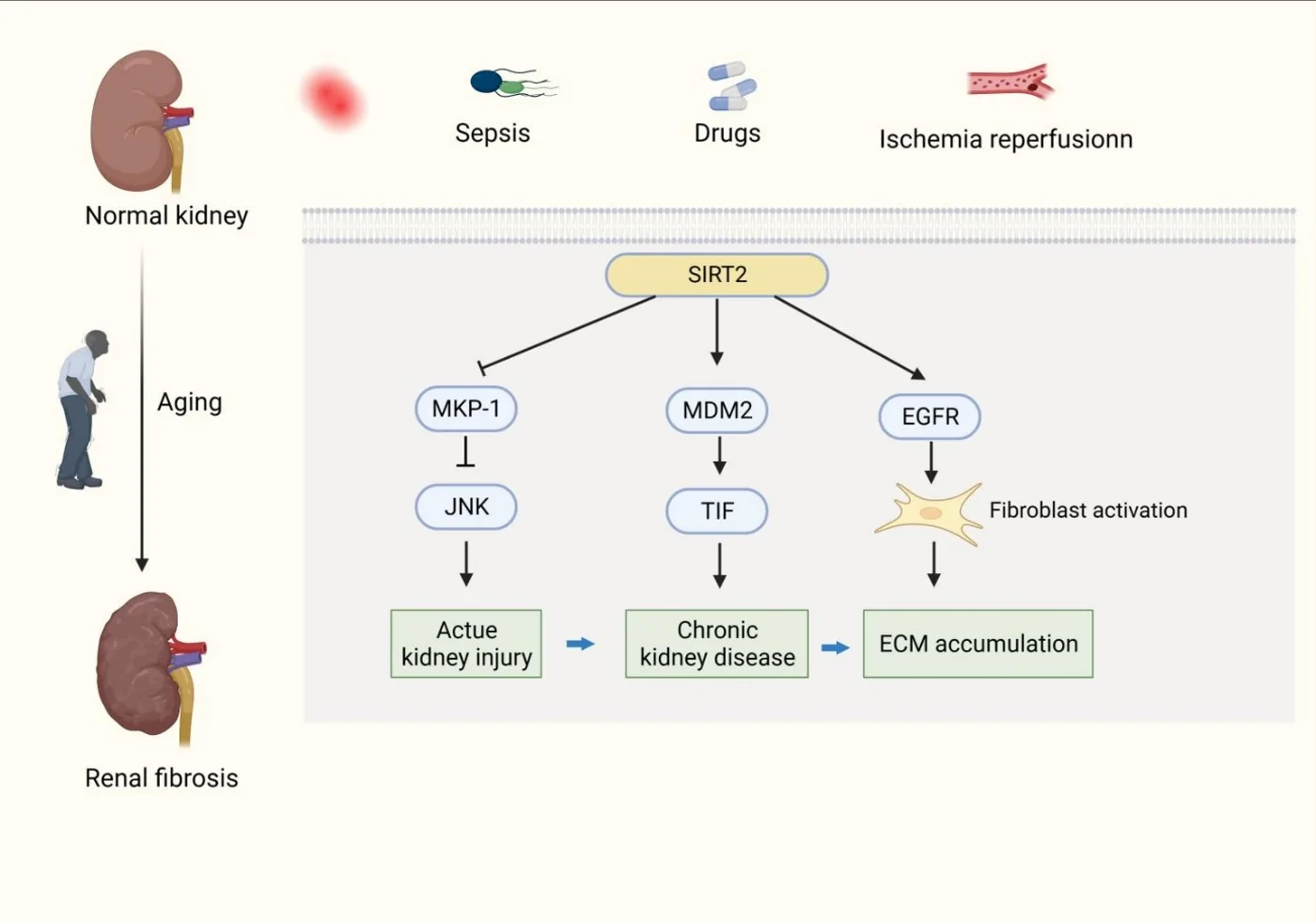
**SIRT2-mediated renal fibrosis**. Acute kidney injury occurs in normal kidneys in response to sepsis, drugs, and ischemia-reperfusion, followed by progression to chronic kidney disease and ultimately renal fibrosis. SIRT2 regulates renal fibrosis by acting on MAPK, MDM2, and EGFR. ECM: extracellular matrix.

The kidney is an important excretory organ in the body, maintaining internal homeostasis through the excretion of metabolic waste, regulation of body fluids, and secretion of endocrine hormones. Renal fibrosis is a chronic and progressive process that affects kidneys in aging and chronic kidney disease (CKD). It is also a worsening process in many types of end-stage renal disease. Recurrent acute kidney injury leads to CKD, and the terminal pathology of chronic kidney disease is renal fibrosis, which is characterized by glomerular sclerosis and tubulointerstitial fibrosis (TIF) [137]. Renal TIF primarily progresses due to tubular epithelial-mesenchymal transition (EMT) and fibroblast activation, accompanied by inflammatory cell infiltration and myofibroblast accumulation [138]. Cellular senescence is prevalent in acute kidney injury and many chronic kidney diseases. Increase of senescent cells elevates the expression of aging-associated cytokines p16 and p21, which further develops acute kidney injury and chronic kidney diseases into renal fibrosis [139]. Renal fibrosis is characterized by EMT of tubular epithelial cells (TEC), during which p21-mediated G2 cell cycle arrest occurs. Concurrently, TGF-β1 induces cell cycle arrest in the G2 phase, limiting the kidney’s repair and regeneration potential, thus exacerbating chronic fibrosis [140]. TGF-β serves as the main driving force of fibrosis progression in renal fibrosis, with epidermal growth factor (EGF) and platelet-derived growth factor (PDGF) acting as co-contributors, which have an important synergistic effect in the development of renal fibrosis. Blocking the TGF-β pathway or targeting EGF and PDGF receptors can prevent renal fibrosis [141]. SIRT2 regulates renal fibrosis by affecting acute renal injury, tubulointerstitial fibrosis, and fibroblast activation (Fig. 3).

### 5.1. SIRT2 promotes acute kidney injury

Acute kidney injury (AKI), a common clinical disease and its severity is closely correlated with subsequent progression to chronic kidney disease (CKD). The etiology of Acute kidney injury (AKI) is complex, including sepsis, medications, and ischemia-reperfusion. Tubular epithelial cells are essential to AKI to CKD transition. In the G2/M phase of EMT, cell cycle arrest and heightened production of pro-fibrotic factors induce tissue fibrosis and renal tubule senescence. Animal studies have found that in 3 different AKI models, senescent cells increase in the proximal tubules of mice, and drive the progression of kidney injury and fibrosis through cellular autonomic activation of innate immune signals and non-cellular autonomic control of pericellular fate [142]. CCL2 is a SASP component identified as a paracrine aging mediator [16]. During aging, SIRT2-mediated FOXO3a deacetylation promotes FasL expression, triggering apoptosis during renal ischemia-reperfusion [143]. In addition, SIRT2 regulates LPS-induced tubular CXCL2 and CCL2 expression. SIRT2 deficiency improves LPS-induced neutrophil and macrophage infiltration, acute tubular injury, and renal function. Mitogen-activated protein kinase phosphatase-1 (MKP-1) activation is a doubly specific protein phosphatase that regulates mitogen-activated protein kinase (MAPK) signaling. During the interaction of SIRT2 with MKP-1, SIRT2 knockdown increases the acetylation of MKP-1 and inhibits the phosphorylation of p38 MAPK and c-Jun N-terminal kinase (JNK) in LPS-treated MPT cells [144]. Activation of both C-Jun JNK and p38 MAPK were identified in cisplatin-induced kidney injury. SIRT2 is associated with cisplatin-induced kidney injury by regulating MKP-1 expression [145].Thus silencing SIRT2 may be an important treatment option for AKI. In elderly people, aging can cause changes in kidney structure and function, increase susceptibility to various damaging factors, and significantly increase the incidence of acute kidney injury and chronic kidney disease compared to the general population. Therefore, studying the characteristics of SIRT2 in kidney aging and the mechanism of fibrosis can effectively prevent and treat elderly kidney diseases, reduce mortality, and improve prognosis.

### 5.2. SIRT2 promotes renal TIF

Renal tubulointerstitial fibrosis (TIF) is the symbol of chronic kidney disease, which eventually develops into renal fibrosis. The main mechanisms of renal TIF progression include tubular EMT and fibroblast activation. TGF-β1 has been considered as the central mediator of tissue fibrosis across multiple organs, including skin, liver, kidneys and lungs [146]. TGF-β1 is a major regulator of myofibroblast differentiation in renal fibrosis. Senescent renal epithelial cells produce multiple components of the SASP, with TGF-β1 inducing fibrosis and inhibiting the proliferation of injured renal tubules. Elevated SIRT2 levels have been identified in the renal tubule interstitial of TIF patients and UUO mice. Concurrently, TGF-β1-induced activation of renal interstitial fibroblasts boosts SIRT2 expression [11]. The ubiquitin E3 ligase MDM2 mediates fibroblast activation and renal TIF [147]. Positioned as an upstream regulator of MDM2, SIRT2 inhibitors inhibit TGF-β1-induced MDM2 up-regulation. Furthermore, TGF-β1 participates in the aging of glomerular endothelial cells and up-regulates the expression of p16/p21, thereby further promoting renal fibrosis [148].

### 5.3. Inhibition of SIRT2 can inhibit fibroblast activation

ECM accumulation resulting from fibroblast activation is a necessary condition for TIF that can culminate in renal fibrosis. Epidermal growth factor receptor (EGFR) [149] and platelet-derived growth factor receptor β(PDGFRβ) [150] are two major cell surface receptors involved in the activation and proliferation of renal fibroblasts. Activation of EGFR and PDGFRβ may promote renal fibrosis. The PDGFR-β-mediated pathway is inhibited in senescent cells [151]. In senescent kidneys, changes in vascular status via the EPCR pathway influence fibrosis development [152]. SIRT2 is expressed in cultured renal interstitial fibroblasts (NRK-49F), and the SIRT2 inhibitor AGK2 blocks EGFR and PDGFRβ phosphorylation, thus inhibiting the renal fibroblast activation and cell proliferation [153]. SIRT2 promotes renal fibrosis by mediating TIF and fibroblast activation, making SIRT2 inhibitors promising therapeutic agents.

## 6. SIRT2 influences cardiac fibrosis during aging

**Figure 4.**
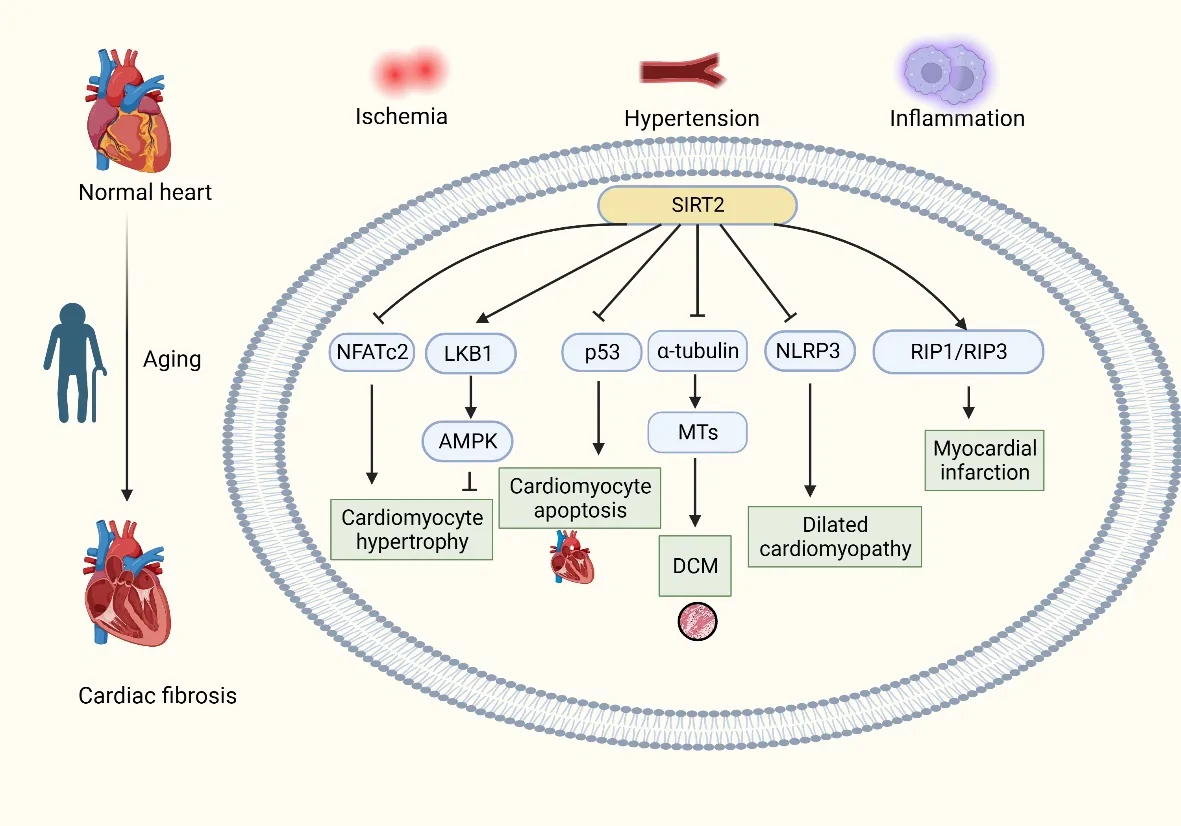
**SIRT2-mediated cardiac fibrosis**. During aging, the heart develops fibrosis accompanied by local ischemia, hypertension, and inflammation. In pathological cardiac hypertrophy, SIRT2 inhibits its development by inhibiting NFATc2. On one hand, SIRT2 promotes cardiac hypertrophy by promoting AMPK activation through the deacetylation kinase LKB1. On the other hand, SIRT2 promotes DCM by inhibiting P53, α-tubulin, NLRP3 and promoting RIP1/3. DCM: Dilated cardiomyopathy.

The heart is the muscular organ of the blood vessel system and the circulatory center of the body, providing systemic blood distribution throughout the body. Cardiac fibrosis, a pathological condition that occurs after injury and during aging. Fibroblasts play an important role in depositing collagen and other ECM components during cardiac fibrosis. Fibrosis is the common feature of many acute and progressive heart diseases [154]. Various cardiovascular diseases, including coronary heart disease, hypertrophy remodeling that is caused by hemodynamic overload (pressure or volume) and cardiomyopathy are associated with fibrosis. Stimulation of myofibroblast activation is the etiology of different injuries, including ischemia, overload and diabetic cardiomyopathy (DCM). Factors like TGF-β, mechanical stress, and other growth factors facilitate this transdifferentiation [155]. Oxidative stress, Toll-like receptor signaling, and pro-inflammatory cytokines have been implicated in the expression of TGF-β subtypes in fibrotic tissues. The MAPK pathway mediates the AngII/AT1R effect. P38 MAPK, a member of MAPK, has been shown to play a central role in promoting the expression of pro-fibrotic genes in fibroblasts [156]. Another key factor controlling the fibrotic response is the renin-angiotensin-aldosterone system (RAAS) and its effector Ang II. It promotes fibrosis and the up-regulation of EMC proteins in the heart and blood vessels by activating AT1R [157].Aging is a major risk factor for various heart diseases, such as left ventricular hypertrophy, myocarditis, and coronary heart disease. Cellular aging plays an important role in the pathophysiology of age-related heart disease. Understanding the mechanisms of various cardiac cellular aging and the relationship between SIRT2 and cardiac fibrosis is beneficial for fundamentally exploring new treatment methods to prevent or treat age-related heart diseases (Fig. 4).

### 6.1. SIRT2 prevents heart hypertrophy

Aging-associated cardiac fibrosis is characterized by cardiomyocytes hypertrophy, which is the primary response of the heart to hemodynamic overload (such as stress or volume overload). This response is marked by amplified protein synthesis and fibroid reorganization, resulting in an increase in cardiomyocyte size. SIRT2 can bind to the Nuclear Factor of Activated T Cells 2(NFATc2) and deacetylate it, further repressing the occurrence of pathological cardiac hypertrophy [158]. SIRT2 promotes AMPK activation by deacetylating the kinase LKB1. Loss of SIRT2 reduces AMPK activation, promotes age-associated and angiotensin II (Ang II) -induced cardiac hypertrophy, and attenuates metformin-mediated cardioprotective effects [91]. Epigenetic regulation is basically involved in the development of cardiomyocyte hypertrophy. In Ang II -induced cardiomyocyte hypertrophy, PHD protein 19 (PHF19) fosters cardiac hypertrophy by epigenetic inhibition of SIRT2 expression [159]. The constitutive photomorphic 9 (COP9) semaphone complex subunit 6 (CSN6) exacerbates Ang II-induced cardiomyocyte hypertrophy by inhibiting SIRT2 [160]. A novel circ_0018553 modulates miR-4731/ SIRT2 signaling pathway to prevent angiotensin-induced cardiomyocyte hypertroph [161]. These findings underline the positive role of SIRT2 against pathologic myocardial hypertrophy, indicating SIRT2 as a potential therapeutic intervention target for aging and stress-induced cardiac hypertrophy, which can effectively inhibit the occurrence and development of pathological cardiac hypertrophy.

### 6.2. SIRT2 and myocarditis

Cardiac aging is often accompanied by inflammation and fibrosis. Myocardial cell inflammation is an important link in the progression of fibrosis. SIRT2 has different functions in myocarditis. On one hand, the activation of Toll-like receptor 4 (TLR4) is a vital regulator of inflammation. TLR4 reduces the expression and activity of SIRT2, increasing the p53 acetylation and promoting cardiomyocyte apoptosis [162]. Over-expression of LncHrt protects the heart from myocardial infarction, with SIRT2 acting as an LncHrt interacting protein in cardiometabolic regulation. Mechanistically, LncHrt maintains SIRT2 activity via CDK5, which then deacetylates LKB1 and enhances LKB1 phosphorylation, ultimately activating the LKB1-AMPK signaling [163]. Protein glycosylation of advanced glycosylated end products (AGEs) accumulated in the myocardium, which is a major cause of diabetic cardiomyopathy (DCM). Microtubules (MTs) are cytoskeletal filaments and SIRT2 is involved in the deacetylase of MT during DCM. Age-induced MT stability in cardiomyocytes is mediated via perturbations in the SIRT2/ acetylated α-tubulin signaling pathway. Studies have shown that AGEs and its receptors promote the development of DCM in cardiomyocytes by inhibiting the SIRT2/ acetylated α-tubulin signaling pathways, bolstering the stability of MT [164]. Colchicine, a potent anti-inflammatory agent, up-regulates SIRT2 expression, resulting in NLRP3 inflammatory body inactivation through NLRP3 deacetylation. Colchicine improves dilated cardiomyopathy through SIRT2-mediated inhibition of NLRP3 inflammasome activation [165]. On the other hand, death ligands such as tumor necrosis factor TNF-α, activate necrosis by stimulating the formation of complexes containing receptor-interacting protein 1 (RIP1) and receptor-interacting protein 3 (RIP3). Pharmacological inhibition of SIRT2 blocks TNF-α-induced cell necrosis, suggesting that SIRT2 inhibitors may constitute a novel approach to prevent necrotic injury, including ischemic stroke and myocardial infarction [166].

## 7. SIRT2 affects fibrosis of other organs during aging

In the aging process, SIRT2 is implicated in both pulmonary fibrosis and vascular fibrosis. Pulmonary fibrosis (PF), a chronic interstitial lung disease, is usually correlated with aging. Aging drives oxidative stress and abnormal inflammation, promoting the trans differentiation of fibroblasts into myofibroblasts, resulting in excessive deposition of ECM in the interstitum [167]. Studies have shown that SIRT2 enhances allergic asthma inflammation by stimulating eosinophilic recruitment, pro-inflammatory chemokine accumulation, and goblet cell proliferation [168]. Meanwhile, SIRT2 inhibitor AGK2 improves mast cell-mediated allergic airway inflammation and fibrosis by inhibiting IgE receptor (FcεRI) FcεRI/TGF-β signaling pathway [169]. SIRT2 is involved in the development of idiopathic pulmonary fibrosis (IPF) by regulating the Smad2/3 pathway. Vascular aging involves the aging process of endothelial and vascular smooth muscle cells, cardiovascular lesions promote fibrosis, pharmacological inhibition of SIRT2 reduces oxidant-induced cytotoxicity in endothelial cells [170], and prevents vascular endothelial cell damage induced by high glucose (HG) through inhibiting p53 and NF-κB signaling pathways [171]. In addition, SIRT2 participates in angiotensin II-induced endothelial cell migration and emerges its role in hypertension-induced vascular remodeling [172].

## 8. Discussion and Prospect

Sirtuins have been shown to play an important role in the homeostasis of tissues and organs, as well as regulating cellular aging. Across different experimental conditions and animal models, the activity of Sirtuins is complex because of their interaction with various genes. Current investigations of SIRT2 and fibrosis development in the aging process involve liver fibrosis, kidney fibrosis, cardiovascular fibrosis, and pulmonary fibrosis. However, the direct literature evidence indicating the function of SIRT2 in age-associated fibrosis remains limited and further research is needed.

A thorough literature review indicates that the SIRT2 protein can be used as a potential therapeutic target for fibrosis treatment. There is substantial evidence suggesting the key contribution of SIRT2 to the occurrence and progression of liver, kidney, and cardiac fibrosis during aging. In the liver, SIRT2 regulates liver fibrosis by affecting hepatic stellate cell's activation, HBV replication, transcription, and non-alcoholic fatty liver disease. In the kidney, SIRT2 regulates renal fibrosis by affecting acute kidney injury, renal tubulointerstitial fibrosis, and fibroblast activation. In the heart, SIRT2 potentially modulates cardiac fibrosis by affecting cardiac hypertrophy and myocarditis. The use of SIRT2 inhibitor in specific liver injury model and kidney injury model showed promising antifibrotic activity, highlighting SIRT2 as an effective therapeutic target for treating fibrotic disease. In diverse fibrosis models, the mechanism of SIRT2 action differs based on different substrates. Given that SIRT2 plays different roles in the development of fibrosis in different organs during aging, comprehensive studies are needed to understand the mechanism of SIRT2 activity in order to develop anti-fibrosis therapies targeting SIRT2.
